# Development of a Flexible Integrated Self-Calibrating MEMS Pressure Sensor Using a Liquid-to-Vapor Phase Change

**DOI:** 10.3390/s22249737

**Published:** 2022-12-12

**Authors:** Yuhong Kang, Scott Mouring, Albrey de Clerck, Shuo Mao, Wing Ng, Hang Ruan

**Affiliations:** 1 Nanosonic, Inc., Pembroke, VA 24136, USA; 2 Department of Mechanical Engineering, Virginia Polytechnic Institute and State University, Blacksburg, VA 24061, USA

**Keywords:** self-calibrating, flexible, phase change, two phase, pressure sensor, MEMS

## Abstract

Self-calibration capabilities for flexible pressure sensors are greatly needed for fluid dynamic analysis, structure health monitoring and wearable sensing applications to compensate, in situ and in real time, for sensor drifts, nonlinearity effects, and hysteresis. Currently, very few self-calibrating pressure sensors can be found in the literature, let alone in flexible formats. This paper presents a flexible self-calibrating pressure sensor fabricated from a silicon-on-insulator wafer and bonded on a polyimide substrate. The sensor chip is made of four piezoresistors arranged in a Wheatstone bridge configuration on a pressure-sensitive membrane, integrated with a gold thin film-based reference cavity heater, and two thermistors. With a liquid-to-vapor thermopneumatic actuation system, the sensor can create precise in-cavity pressure for self-calibration. Compared with the previous work related to the single-phase air-only counterpart, testing of this two-phase sensor demonstrated that adding the water liquid-to-vapor phase change can improve the effective range of self-calibration from 3 psi to 9.5 psi without increasing the power consumption of the cavity micro-heater. The calibration time can be further improved to a few seconds with a pulsed heating power.

## 1. Introduction

In recent years, with the growth of microelectromechanical systems (MEMS) technology, researchers have developed and produced various MEMS-based pressure sensors for a wide variety of engineering applications, such as industrial, chemical, biomedical, aerospace, and automotive uses [1,2,3]. There are mainly five mechanisms used for pressure sensing, including piezoelectric, piezoresistive, capacitive, resonance, and optical. Thanks to CMOS compatible cleanroom mass production capability, high sensitivity, high linearity, high reliability and small size piezoresistive MEMS-based pressure sensors have been the most popular option in the past decade, with continuously improved performances [4].

In a typical piezoresistive MEMS pressure sensor, silicon-based piezoresistors are carefully designed and arranged in a Wheatstone bridge configuration on a pressure-sensitive membrane. Once an external pressure is applied to the membrane, the flexure-induced stress on the piezoresistors causes a resistance change, then transforms into a voltage change by the Wheatstone bridge to improve sensitivity and mitigate temperature dependence. This voltage change is proportional to the applied pressure and can be directly converted to an exact pressure once correctly calibrated. Among the latest research works, many studies focused on enhancing sensitivity with different methods and materials [5,6,7,8]. Meanwhile, another popular topic is mitigating temperature dependence through circuit compensation and other approaches [9,10,11], which makes the in-situ calibration ideal for field monitoring applications.

Recently, flexible pressure sensors and arrays have attracted significant attention since the capability to be attached to or embedded into conformal surfaces is of paramount importance in many applications, such as fluid dynamic studies, structural health monitoring, and wearable sensors [12,13]. Luo et al. developed a cuffless blood pressure monitoring device by building a carbon black-decorated fabric flexible piezoresistive sensor with three epidermal electrocardiogram electrodes [14]. Yu et al. adopted an interesting 3D conductive network to allow their sensor to be both flexible and highly sensitive (136.8 kPa^−1^) at very low pressure (<200 Pa) [15]. Li et al. developed a flexible capacitive pressure sensor based on a dual-structured nanofiber membrane to achieve high sensitivity (0.28 kPa^−1^) in the low-pressure region (0−2 kPa) [16]. Along with other similar research works [17,18,19,20,21,22,23], researchers have shown the significant potential of the flexible pressure sensor. However, most of these sensors may encounter issues in areas such as reliability, repeatability, and hysteresis.

The sensing range of a pressure sensor is generally limited by its calibration range [24], since calibration is essential to provide accuracy. Pressure sensors are usually calibrated by relating the sensor’s output to a known range of input pressures, creating a calibration curve. Typically, the calibration process requires expensive equipment and the sensor to be removed from the use site. This leads to costly periods of extended downtime, especially for semi-permanently installed embedded sensors. Furthermore, even after proper calibration, mechanical wear from repeated use causes sensor inaccuracies, such as hysteresis, nonlinearity, and signal drift [25]. A low-cost, rapid self-calibration sensor can be a great option to solve these problems.

Contrary to conventional calibration, self-calibration takes place in situ without external input. During the self-calibration process, the sensor creates its own input reference signal, which can be achieved by coupling a pressure sensor with an actuator device that simulates an applied, known pressure. Common actuation types include mechanical, electrical, and thermal [26]. Using an actuator paired with a sensor is an established method for self-testing sensors [27]. The reference signal is then compared to the sensor reading to construct a calibration curve.

Currently, very few self-calibrating pressure sensors can be found in the literature. Yameogo et al. developed a wireless, piezoresistive pressure sensor for in-vivo biomedical applications [28]. They sent an adjustable voltage between two electrodes, causing the sensor membrane to deflect and act as a reference pressure for self-calibration. Another self-calibrating pressure sensor found in the literature was developed by MKS Instruments, Inc., using a multi-sensor system, with one pressure sensor and one reference sensor that share a common sealed chamber [29]. Electrostatic actuation can deflect the pressure-sensitive membrane of the reference sensor, which is used to correct drift caused by aging of the pressure sensor. However, using multiple sensors in a single unit to accomplish self-calibration can be cost-ineffective and counterproductive.

Therefore, the authors have developed a smart, flexible MEMS piezoresistive pressure sensor capable of self-calibration, presented in previous publications [30,31]. In the enclosed side of the sensor membrane, the system heats the air to increase the cavity pressure (referred to as the “single-phase sensor” below). Then the internal temperature is measured by a thermistor to calculate the internal pressure, which is compared with the sensor output. Previous modeling work by the authors has shown that the cavity heater could result in a 3 psi increase in the sensing cavity, but 95% of the heat generated was lost to conduction through the substrate. In this study, to extend the calibration range and increase the power efficiency, the authors introduced a liquid-to-vapor phase change in the sensing cavity, since evaporation leads to a significant pressure rise (referred to as the “two-phase sensor” below). Deionized water was chosen for the cavity liquid due to its availability, low corrosivity, low conductivity, and predictable phase-change behavior [32,33].

The only other MEMS pressure sensor in the literature that incorporated a liquid-to-vapor phase change within a sealed reference cavity was developed by Huo et al. [34]. The sensor heated water in the sensing cavity to test if the sensor was functioning. However, unlike the presented sensor, this sensor did not measure the internal cavity temperature. Therefore, it was unable to self-calibrate to resolve hysteresis and drift.

In this paper, the authors discuss the development of an innovative self-calibrating flexible pressure sensor. With the help of the cavity fluid’s phase change, this flexible sensor can self-calibrate over a large range with small power consumption, extending its operating range and mitigating the heating’s effect on sensor performance. First, the authors established the physics of a liquid-to-vapor phase change in a closed domain. Then CFD modeling was used to investigate the pressure and temperature distribution in the sensor cavity. Finally, the authors built a prototype sensor based on the analytical and computational results and compared its performance with expected values, proving the feasibility and practicability of this sensor design.

## 2. Modeling and Development

### 2.1. Governing Physics

When heating multiple gases in a closed container, Dalton’s law of partial pressures can be used to calculate the total pressure of the gas mixture. As the water in the reference cavity heats up, vapor and air will coexist in the cavity. According to Dalton’s law,

(1) $$P_{T}=P_{a}+P_{v}$$

where *P_T_* is the absolute total pressure in the cavity, and *P_a_* and *P_v_* are the absolute partial pressures of the air and vapor, respectively.

In a closed container with water and air of known volumes and initial states, the partial pressure can be calculated using the ideal gas law,

(2) $$P_{a,f}=\frac{P_{a,i}T_{a,f}}{T_{a,i}}$$

where *P_a,i_* and *T_a,i_* are the initial absolute partial pressure and temperature of the air in the cavity, respectively, and *P_a,f_* and *T_a,f_* are the final absolute partial pressure and temperature of the air in the cavity after heating, respectively.

For closed systems containing both an air–water vapor mixture and liquid water, the partial pressure of the water vapor in the mixture is equal to the saturation pressure of water at the temperature of the air–water vapor mixture [35]. Antoine’s equation is often used to approximate the vapor pressure of water as a function of temperature in the form of the piecewise function

(3) $$P_{v}=10^{8.07131-\frac{1730.63}{233.426+T}},\;1\;{}^{\circ}C\;\leq \;T\leq \;100\;{}^{\circ}C$$

(4) $$P_{v}=10^{8.14019-\frac{1810.94}{244.485+T}},\;100\;{}^{\circ}C\;\leq \;T\leq \;374\;{}^{\circ}C$$

where *P_v_* is the water vapor pressure in mmHg and *T* is the temperature of the water in degrees Celsius [36]. Summing the equations for the partial pressures of the air and water vapor produces the equation for the theoretical total absolute pressure in the sealed reference cavity,

(5) $$P_{T}=\frac{P_{a,i}T_{a,f}}{T_{a,i}}+10^{8.07131-\frac{1730.63}{233.426+T}},\;1\;{}^{\circ}C\;\leq \;T\leq \;100\;{}^{\circ}C$$

(6) $$P_{T}=\frac{P_{a,i}T_{a,f}}{T_{a,i}}+10^{8.14019-\frac{1810.94}{244.485+T}},\;100\;{}^{\circ}C\;\leq \;T\leq \;374\;{}^{\circ}C$$

**Figure 1:**
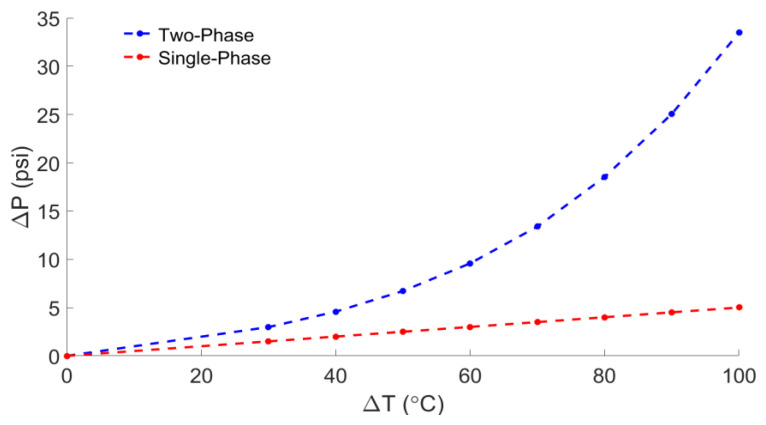
Calculated pressure change as a function of the temperature change inside a rigid, closed container for single-phase (air only) and two-phase (water–air mixture) regimes.

Figure 1 below compares the pressure change, according to Equations (5) and (6), with (a) both air and water and (b) air only. With a temperature change of 100 °C, the phase change potentially quadruples the effective range of the sensor’s self-calibrating capabilities. A CFD model of the sensor cavity was developed and will be discussed later to investigate the potential temperature and pressure gradients in the cavity after a liquid-to-vapor phase change.

### 2.2. Sensor Cavity Two-Phase Modeling

To further confirm the feasibility of this two-phase calibration concept, a steady-state 2D Ansys Fluent model was developed to guide the design of the sensor prototype. The CFD model was solved with the Reynolds-Averaged Navier Stokes (RANS) equations using the commercial CFD software ANSYS Fluent 2021 (Ansys, Inc., Canonsburg, PA, USA). The simulation adopted a steady-state pressure–velocity coupling algorithm with the RNG k-ε turbulence model using a scalable wall function. 

**Figure 2:**
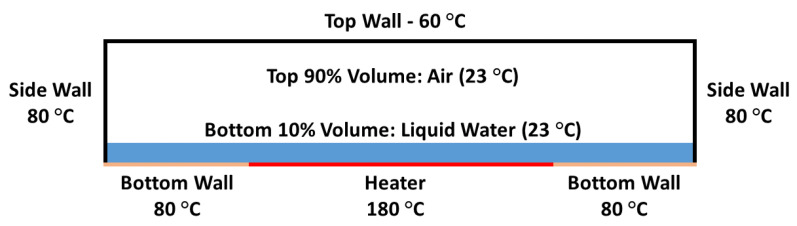
Initial and boundary conditions for the 2D two-phase sensor cavity Ansys Fluent model.

The sensor cavity, whose boundaries were assumed rigid, was selected as the flow field in the simulation. The fluid domain dimensions matched the rectangular side profile of the self-calibrating sensor’s 2 mm × 0.29 mm reference cavity. It was then divided into a mesh consisting of 150 k rectangular elements, while a mesh independence study suggested that finer meshes only lead to a change of less than 5% in temperature and pressure. In addition, all the boundaries were set to match the measured operating temperatures at the heater power of 0.45 W, according to previous Ansys modeling work [31], as shown in Figure 2.

In the flow field, three working fluids were defined as the cavity fluid: ideal-gas air, ideal-gas water vapor, and liquid water, while a Volume of Fluid (VoF) model described the water–vapor phase change. At the start of the simulation, the bottom 10% volume of the cavity was room temperature liquid water, and the other 90% was filled with room temperature air.

**Figure 3:**
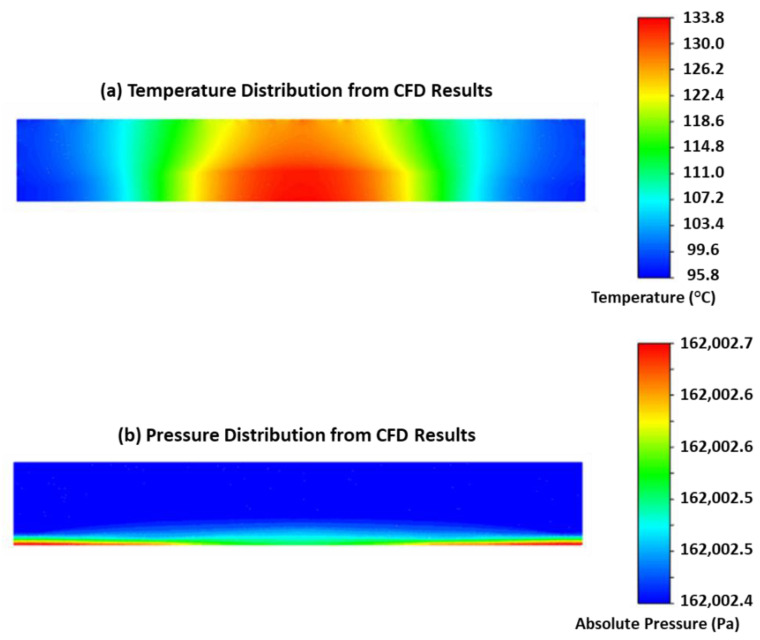
Two-dimensional sensor cavity simulation results of (**a**) temperature and (**b**) absolute total pressure.

Figure 3 shows the temperature and pressure distributions of the steady-state results. In the temperature contour plot in Figure 3a, there is a large temperature gradient in the sensing element’s cavity, similar to what was seen in the previous thermal modeling of the single-phase sensor [31]. Consequently, the location of the cavity thermistor will affect the calibration accuracy and needs to be carefully selected. Figure 3b shows that the internal cavity pressure distribution is uniform, which means the piezoresistors will be applied nearly identical pressures and allow the membrane to function normally. With the guidance provided by the 2D cavity Ansys Fluent model, the first two-phase, self-calibrating pressure sensor prototype was developed.

## 3. Two-Phase Sensor Prototype

### 3.1. Development and Description

**Figure 4:**
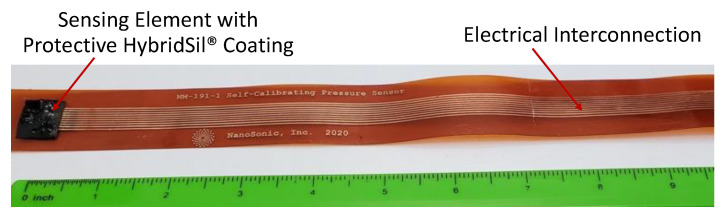
Photo of a prototype flexible two-phase, self-calibrating pressure sensor.

A prototype flexible two-phase, self-calibrating pressure sensor, shown in Figure 4, was fabricated with a design similar to that used for the previous single-phase sensor. The sensor prototype uses a novel flexible strip packaging, which allows the sensor to be mounted to contoured surfaces, such as aircraft nosecones or the exterior of underwater vehicles, while maintaining a low profile. On the far left end of the flexible strip housing is the two-phase sensing element, which is housed underneath NanoSonic’s protective HybridSil^®^ (NanoSonic, Inc., Pembroke, VA, USA) coating for anticorrosion, anti-abrasion and hydrophobicity applications.

**Figure 5:**
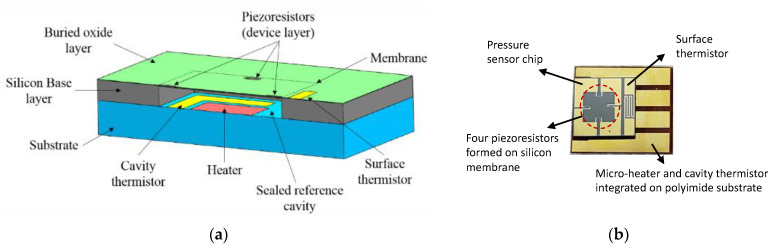
(**a**) Cross-section view of the sensing element inside the self-calibrating pressure sensor and (**b**) a microscopic image of such a sensor chip, note that the red circle indicates the sensing region with four piezoresistors built on the membrane [31].

The self-calibrating pressure sensor’s sensing element, shown in Figure 5, has a 4 mm square base and a total height of 405 μm. Pressure sensors are fabricated from a silicon-on-insulator (SOI) wafer, which consists of a thin silicon device layer, a buried oxide layer (BOX), and a silicon base or handle layer. A hollow reference cavity is carved out of the silicon base layer using deep reactive-ion etching (DRIE). On the device layer, four piezoresistors are arranged in a Wheatstone bridge configuration with gold electrical interconnection lines and pads for packaging. Before the polyimide substrate is bonded to the pressure sensor chip to create the sealed reference cavity, both the gold thin film-based cavity heater and thermistor are manufactured directly onto the substrate surface. With the heater enclosed with the thermistor in the sealed reference cavity, a controllable thermopneumatic actuator is formed, giving the sensor self-calibrating capabilities [31]. As the heater heats the fluid in the reference cavity, the fluid evaporates and expands, deforming the membrane and the device layer. In addition, a surface thermistor fabricated beside one of the pressure-sensing elements monitors the device layer’s temperature.

To create the two-phase sensor, a small water deposit was added to the sealed reference cavity of the sensing element shown in Figure 5. Liquid water was added to the cavity using a micropipette with a minimum volume of 0.1 μL. The total amount of water added was about 10% of the cavity’s total volume. 

### 3.2. Testing and Calibration Procedures

**Figure 6:**
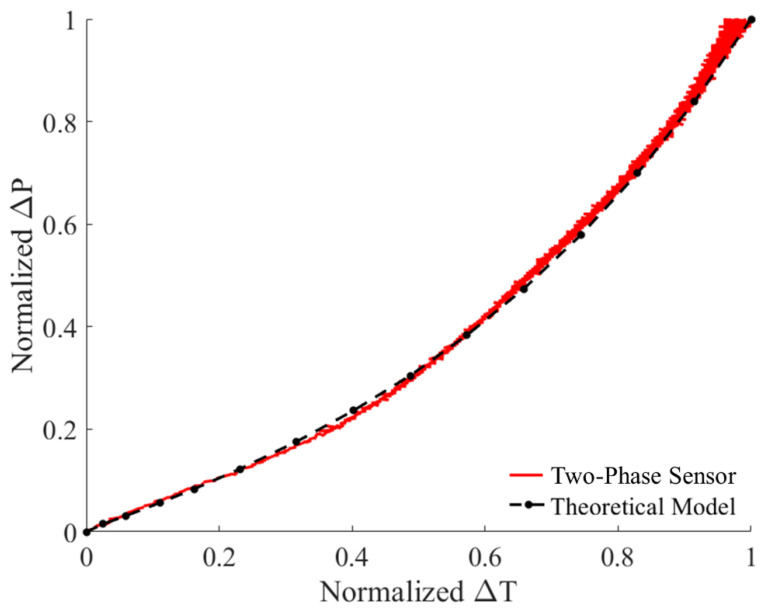
Normalized pressure and temperature change measurements for the prototype sensor compared to theoretical results.

Before calibrating the prototype two-phase sensor, a shakedown test was performed to confirm that liquid water was successfully sealed in the reference cavity during sensor fabrication. To confirm the presence of the phase change mechanism, the measured pressure change and cavity temperature change were normalized as Δ*X_n_* using the equation

(7) $$\Delta X_{n}=\frac{\Delta X-\Delta X_{\min}}{\Delta X_{\max}-\Delta X_{\min}}$$

where Δ*X* is either the cavity pressure or temperature change, and Δ*X*_min_ and Δ*X*_max_ refer to the minimum and maximum value of Δ*X*, respectively. The results were compared to the normalized theoretical pressure change according to Equations (5) and (6), as shown in Figure 6. The two overlapping nonlinear curves proved the existence of a water liquid-to-vapor phase change in the sensor cavity.

After confirming the presence of liquid water in the sensor cavity, the sensor was pressure calibrated at room temperature. During the calibration, the two-phase sensor was placed in a sealed chamber connected to a pressure-control handpump and inside a temperature-controlled environmental chamber set to 20 °C. The heating element in the cavity was kept off for the entirety of the pressure calibration to ensure the measured pressure was only applied by the hand pump. Using the hand pump, the pressure in the sealed chamber was varied from −10 psi to 10 psi in 2 psi increments, providing the data necessary to create a linear fit equation to convert the sensor pressure voltage signal to a pressure (psi). Since the measured signal from an externally applied pressure is equal and opposite to that from an internal cavity pressure of the same magnitude, this calibration curve can be used to determine the pressure inside the sensor cavity. Then the exact pressure resulting from the liquid-to-vapor phase change inside the reference cavity can be determined.

## 4. Results

**Figure 7:**
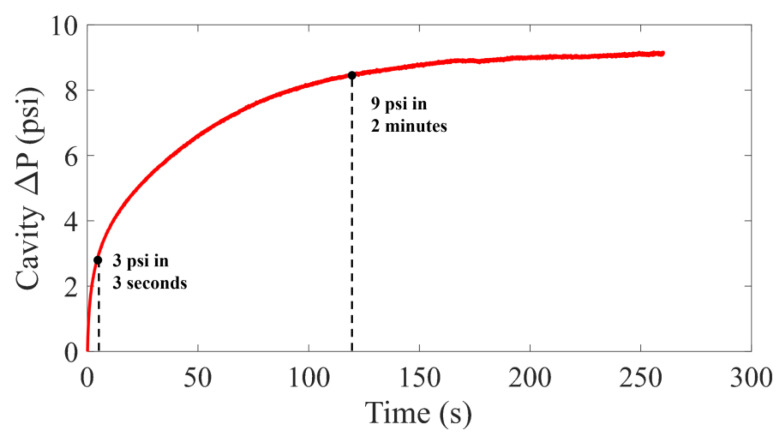
Measured cavity pressure after powering on the cavity heater.

During the sensor tests, the cavity heater was turned on to 0.45 W and held until the cavity temperature reached a steady state. Figure 7 shows the resulting cavity pressure as a function of time. Three seconds after turning on the heating element, the cavity pressure in the prototype two-phase sensor increased by 3 psi, reaching the equilibrium pressure change in the single-phase sensor at similar heater power levels [31], but within a much quicker time. The pressure change is increased to 9 psi after just 2 min, tripling the effective range of self-calibration of the sensor. The gauge pressure in the sensor cavity also appears to reach 9.5 psi at the steady state, closely matching the 2D cavity Ansys Fluent model (9.8 psig). The calibration time can be further improved to a few seconds with a pulsed heating power. 

**Figure 8:**
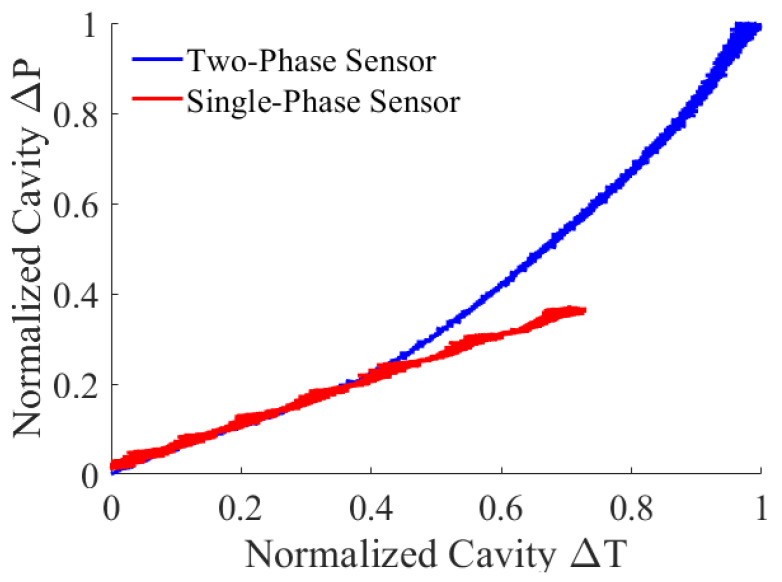
Comparison of the normalized cavity pressure vs. the normalized cavity temperature change for the two-phase and single-phase sensor.

During the heating process, the pressure–temperature relation of the cavity fluid was recorded and compared to the air-only sensor. For direct comparison, the authors have adopted the same normalization method from Equation (7) to illustrate the pressure vs. temperature data from both sensors (i.e., single-phase and two-phase), as shown in Figure 8. The difference was initially insignificant, with a small temperature change since most water remained in the liquid state. As the water was heated up, the two-phase sensor started to show the advantages of higher pressures associated with a liquid-to-vapor phase change. As a result, the two-phase sensor achieved a wider calibration range. In other words, the two-phase sensor can reach the same pressure range with less heater power and a faster response time.

Not only is the performance of the self-calibrating pressure sensor significantly improved by adding a liquid-to-vapor phase change, but the two-phase sensor prototype also has notable advantages over other available self-calibrating pressure sensors. Unlike the presented two-phase sensor, the self-testing pressure sensor developed by Huo et al. was unable to resolve hysteresis and drift (i.e., self-calibrate) due to the lack of an internal cavity temperature measurement [34]. In addition, the outdated fabrication techniques limit the resolution to 0.06 mV/psi, while the presented two-phase self-calibrating sensor fabricated using SOI wafer technology with deep reactive-ion etching (DRIE) is almost 80 times greater at 4.7 mV/psi [31,37,38]. Such a sensor also exhibits a much higher calibrating range (9.5psi) than the self-calibrating sensor developed by Yameogo et al. (~3 psi) [28]. Compared to the multi-sensor self-calibration system, the presented approach has a simpler design and lower fabrication cost [29].

Furthermore, the two-phase sensor in this study uses a fixed volume of liquid in the reference, which allows for repeatable testing and self-calibration. However, the cavity of such a self-testing pressure sensor must be entirely drained before use and refilled again for functionality checks, creating a potential repeatability concern.

## 5. Conclusions and Future Work

Based on the analytical analysis and CFD modeling results, this study successfully designed and fabricated a prototype flexible two-phase, self-calibrating pressure sensor. The sensor’s reference cavity was filled with 10% liquid water by total volume. The phase change mechanism resulted in a cavity pressure change of 9.5 psi, tripling the maximum cavity pressure change of the previous single-phase design at a similar heater power. The two-phase, self-calibrating pressure sensor was also advantageous over other available phase-change-based piezoresistive pressure sensors, presenting a higher resolution and a simpler design.

**Figure 9:**
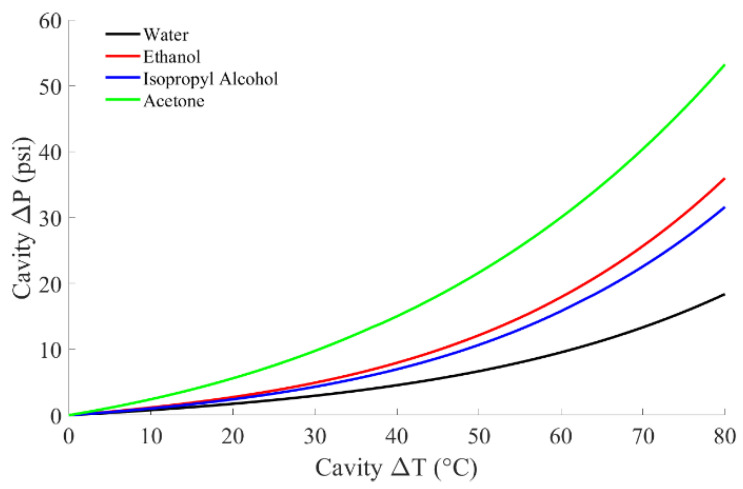
Theoretical pressure change vs. temperature change for multiple different liquids.

Future work will include the investigation of alternative cavity liquids that could provide higher reference pressures without increasing heater power demands to extend the calibration range further. Figure 9 shows the simulated pressure change caused by ethanol, isopropyl alcohol, and acetone compared to water. These liquids were chosen due to their lower saturation temperatures at atmospheric pressure than water, resulting in higher vapor pressures at a given temperature [39].

Further research is also needed to determine whether acetone, isopropyl alcohol, and ethanol will react with silicon, glass, and heater material (Au) over time at high temperatures, causing potential leaks to form in the sensor’s reference cavity. In addition, as the calibration range is extended, the sealing method must be improved to deal with elevated temperatures and pressures.

## Data Availability

The data presented in this study are available on request from the corresponding authors.

## Nomenclature

*a*	Evaporation frequency
*b*	Condensation frequency
** $\dot{m}$ **	Mass flow rate
*P*	Absolute pressure
*T*	Temperature
*α*	Phase volume fraction
*ρ*	Phase density
**Subscripts**	
*a*	air
*f*	final
*i*	initial
*l*	liquid
*sat*	saturated
*T*	total
*v*	vapor
